# Genomic prediction for numerically small breeds, using models with pre-selected and differentially weighted markers

**DOI:** 10.1186/s12711-018-0419-5

**Published:** 2018-10-10

**Authors:** Biaty Raymond, Aniek C. Bouwman, Yvonne C. J. Wientjes, Chris Schrooten, Jeanine Houwing-Duistermaat, Roel F. Veerkamp

**Affiliations:** 1 Animal Breeding and Genomics, Wageningen University and Research, P.O. Box 338, 6700 AH Wageningen, The Netherlands; 2 Biometris, Wageningen University and Research, 6700 AA Wageningen, The Netherlands; 3 CRV BV, P.O. Box 454, 6800 AL Arnhem, The Netherlands; 4 Department of Medical Statistics and Bioinformatics, Leiden University Medical Centre, 2333 ZC Leiden, The Netherlands; 5 School of Mathematics, Faculty of Mathematics and Physical Sciences, University of Leeds, Leeds, LS2 9JT UK

## Abstract

**Background:**

Genomic prediction (GP) accuracy in numerically small breeds is limited by the small size of the reference population. Our objective was to test a multi-breed multiple genomic relationship matrices (GRM) GP model (MBMG) that weighs pre-selected markers separately, uses the remaining markers to explain the remaining genetic variance that can be explained by markers, and weighs information of breeds in the reference population by their genetic correlation with the validation breed.

**Methods:**

Genotype and phenotype data were used on 595 Jersey bulls from New Zealand and 5503 Holstein bulls from the Netherlands, all with deregressed proofs for stature. Different sets of markers were used, containing either pre-selected markers from a meta-genome-wide association analysis on stature, remaining markers or both. We implemented a multi-breed bivariate GREML model in which we fitted either a single multi-breed GRM (MBSG), or two distinct multi-breed GRM (MBMG), one made with pre-selected markers and the other with remaining markers. Accuracies of predicting stature for Jersey individuals using the multi-breed models (Holstein and Jersey combined reference population) was compared to those obtained using either the Jersey (within-breed) or Holstein (across-breed) reference population. All the models were subsequently fitted in the analysis of simulated phenotypes, with a simulated genetic correlation between breeds of 1, 0.5, and 0.25.

**Results:**

The MBMG model always gave better prediction accuracies for stature compared to MBSG, within-, and across-breed GP models. For example, with MBSG, accuracies obtained by fitting 48,912 unselected markers (0.43), 357 pre-selected markers (0.38) or a combination of both (0.43), were lower than accuracies obtained by fitting pre-selected and unselected markers in separate GRM in MBMG (0.49). This improvement was further confirmed by results from a simulation study, with MBMG performing on average 23% better than MBSG with all markers fitted.

**Conclusions:**

With the MBMG model, it is possible to use information from numerically large breeds to improve prediction accuracy of numerically small breeds. The superiority of MBMG is mainly due to its ability to use information on pre-selected markers, explain the remaining genetic variance and weigh information from a different breed by the genetic correlation between breeds.

## Background

The accuracy of genomic prediction (GP) depends on the size of the reference population. Therefore, accuracy of GP is limited in numerically small populations [1, 2]. Potentially, information from numerically larger breeds can be used to predict genomic breeding values (GEBV) of animals in numerically small breeds, through a method called across-breed GP. However, in practice, it has been shown that across-breed GP does not result in significant improvement in prediction accuracy, as compared with within-breed GP [3–6]. In some cases, across-breed GP can result even in negative prediction accuracies [3, 4]. One of the suggested reasons for poor prediction accuracies across breeds is that breeds differ in patterns of linkage disequilibrium (LD) between quantitative trait loci (QTL) and markers. Some studies suggested that increasing marker density, increases the probability that some of the markers are close to the QTL and have a consistent LD across breeds. Therefore, GP across breeds is expected to improve with increasing marker density [7, 8]. In contrast to these expectations, Van den Berg et al. [9] and Raymond et al. [10] showed that simply increasing the density of markers up to whole-genome sequence does not improve accuracy of GP across breeds.

As an alternative to a simple increase in marker density, some studies suggested that across-breed prediction should be based on the QTL themselves, excluding potential non-causal markers [11, 12]. While the use of pre-selected markers or known QTL has been shown to significantly increase the accuracy of across-breed GP [9], the magnitude of the increase in accuracy depends on the similarity of the effects of true QTL between the breeds [13, 14]. This similarity can be measured by the genetic correlation (*r*_g_) between breeds [15, 16], that can be estimated using a multi-breed genomic relationship matrix (GRM) [17, 18]. Accurately pinpointing QTL in the genome is often not trivial, and in most cases, the identified QTL explain only a fraction of the total genetic variance for the traits of interest.

Our hypothesis is that the accuracy of GP in numerically small breeds can be improved using a model that uses all available markers, but weighs markers that have a significant effect on the trait differently from other markers. The model must also account for the *r*_g_ between breeds in the reference and validation breeds, and use it to weigh the contributions of the breeds in the reference population to predict the breeding values for individuals from the breed of interest [17]. The objective of this study was to test a multi-breed multi-GRM model (MBMG) that weighs pre-selected significant markers separately from unselected markers, uses unselected markers to explain the remaining genetic variance that can be explained by markers (RGV_m), and weighs the information of breeds in the reference population by the genetic correlation between the reference and target breeds. To validate the performance of MBMG model in the analysis of real phenotypes, we also implemented the model for the analysis of simulated phenotypes.

## Methods

### Genotype data

For this study, we used data on 595 New Zealand Jersey bulls (NZJ) and 5503 Dutch Holstein bulls (DH), all of which had genotypes for single nucleotide polymorphisms (SNPs) on the Illumina Bovine snp50 beadchip (Illumina Inc., San Diego, CA, USA) with 48,912 SNPs remaining after quality control. These SNPs had at least ten copies of the minor allele in each of the considered populations. In a combined dataset of the DH and NZJ bulls, minor allelic frequencies (MAF) ranged from 0.009 to 0.5. This SNP set will be referred to as 50k. We used two additional sets of SNPs that were pre-selected based on their significance for stature in a meta-genome-wide association analysis (GWAS) analysis using imputed whole-genome sequence [19]. This meta-GWAS was performed on 17 populations from different countries, comprising eight breeds, including Holstein and Jersey. The DH population was included in the meta-GWAS, whereas the NZJ population was not included. The meta-GWAS across the populations identified 24,230 genome-wide significant (*p* < 5 × 10e−8) SNPs in 163 distinct QTL regions spread across 27 autosomes. The first set of pre-selected SNPs were the so-called TOP SNPs, which showed the highest level of significance in each of the 163 QTL regions identified in the meta-GWAS analysis [19]. These TOP SNPs accounted for 13.8% of the phenotypic variance for stature in the meta-GWAS. In our study, some of the TOP SNPs had either a very low MAF or segregated only in one of the two breeds and thus, after quality control, only 133 of these TOP SNPs remained. The second set of pre-selected SNPs were the COJO8 SNPs, which were those that showed independent significant effects (*p* < 10e−8) in the meta-GWAS study. We selected these SNPs using the conditional and joint effect analysis as described in Yang et al. [20] and implemented in the GCTA software [21]. From the 24,230 significant SNPs in the meta-GWAS, 357 COJO8 SNPs were selected. TOP and COJO8 SNPs were selected from imputed whole-genome sequence data, and the 50k SNP set used for our analyses did not contain any TOP or COJO8 SNPs. Sixty-five SNPs overlapped between the COJO8 and TOP SNPs.

For the analysis using real phenotypes, 50k, TOP and COJO8 SNP sets were each fitted, one at a time, with a separate single GRM fitted in the prediction model. SNP sets were evaluated in terms of the estimated genetic parameters and prediction accuracy for stature. Furthermore, the TOP SNPs were combined with the 50k SNPs into a single set (50k + TOP), and the 50k + COJO8 SNP set was a combination of COJO8 and 50k SNPs. These combined sets; 50k + TOP and 50k + COJO8, were fitted via one GRM matrix and evaluated for the estimated genetic parameters and prediction accuracy for stature.

### Empirical phenotypes

For the analysis of real phenotypes, estimated breeding values (EBV) for stature and the effective daughter contributions (EDC), which were the number of daughters on which the EBV were based, were available for all 595 NZJ and 5503 DH bulls. Data were provided by CRV BV (Cooperative Cattle Improvement Organization, Arnhem, The Netherlands). EBV and EDC for stature for all bulls were deregressed to obtain deregressed proofs (DRP) and deregressed effective daughter contributions (dEDC), respectively, according to Calus et al. [22]. To obtain DRP, the deregression procedure, called matrix deregression, corrects for the contribution of information on parents, which also includes information on sibs, to an individual’s EBV. Similarly, dEDC were obtained by removing any EDC that are contributed by relatives in the dataset. The dEDC were used as weights for the DRP in subsequent analyses. Mean dEDC were equal to 52 for DH and 17 for NZJ. In the meta-GWAS [19], 3047 Jerseys from Australia were included. However, Australia and New Zealand do not have a joint genetic evaluation procedure for Jerseys, thus, the DRP of the NZJ bulls do not depend on information that went into the meta-GWAS.

### Simulated phenotypes

To validate the performance of the MBMG model in terms of prediction accuracy and genetic parameter estimation, we also performed a simulation study. Phenotypes for all 595 NZJ and 5503 DH were simulated, using the real genotypes for 49,045 SNPs (50k and TOP SNPs combined) in both breeds. A quantitative trait with a heritability (*h*^2^) of 0.8 in both breeds was simulated. The TOP SNPs (*n* = 133) and 150 randomly selected SNPs were assumed to be causal in both breeds. Allele substitution effects of the causal SNPs were sampled from a bi-variate normal distribution with a mean of 0, a variance of 1, and a correlation of 1, 0.5 and 0.25 between the breeds. Allele substitution effects were sampled independently from the allele frequency of causal SNPs. Therefore, the correlation between allele substitution effects was similar to the correlation between breeding values for performance in DH and NZJ, which is defined as the genetic correlation (*r*_g_) between DH and NZJ. In both breeds, a true breeding value (TBV) for individual *i* was calculated as ∑(*x*_*i*,*j*_ * *a*_*j*_), where *x*_*i*,*j*_ is the genotype of individual *i* at causal locus *j* (coded as 0, 1, 2), and *a*_*j*_ is the allele substitution effect of causal variant *j*. The corresponding phenotype was computed as *TBV*_*i*_ + *e*_*i*_, where *e*_*i*_ is the residual effect of individual *i*, sampled from a standard normal distribution with a mean of 0 and a variance equal to $\sigma_{{a_{k} }}^{2} *\left( {\frac{1}{{h^{2} }} - 1} \right)$, where $\sigma_{{a_{k} }}^{2}$ is the genetic variance of TBV for breed *k*. Simulation of phenotypes was carried out in R [23] and was replicated 100 times.

For the analysis of simulated phenotypes, the following SNP sets were selected:

- *ALL (49,045 SNPs)* we considered this set of SNPs to be the default set, which included 283 causal and 48,762 non-causal SNPs.
- *50k (48,912 SNPs)* this set included the 150 randomly selected causal and 48,762 non-causal SNPs.
- *TOP (133 SNPs)* this set consisted of the 133 causal SNPs that were the TOP SNPs in the empirical analysis, which were identified in the meta-GWAS analysis.
- *TOP + RN (283 SNPs)* this set included 133 TOP SNPs and 150 additional randomly selected non-causal SNPs that represented random noise (RN).
- *CAUSAL (283 SNPs)* this set consisted of all the 283 causal SNPs used in this study.
- *NON CAUSAL (48,762 SNPs)* this set consisted of all non-causal SNPs.

### Statistical models

For both the empirical analysis and simulation, multi-breed GRM were calculated for all SNP sets according to the method proposed by Wientjes et al. [17] as:

$${\mathbf{GRM}} = \left[ {\begin{array}{*{20}c} {\frac{{{\mathbf{Z}}_{\text{DH}} {\mathbf{Z}}_{\text{DH}}^{\prime } }}{{\sum 2p_{{{\text{DH}}j}} \left( {1 - p_{{{\text{DH}}j}} } \right)}}} & {\frac{{{\mathbf{Z}}_{\text{DH}} {\mathbf{Z}}_{\text{NZJ}}^{\prime } }}{{\sqrt {\sum 2p_{{{\text{DH}}j}} \left( {1 - p_{{{\text{DH}}j}} } \right)} \sqrt {\sum 2p_{{{\text{NZJ}}j}} \left( {1 - p_{{{\text{NZJ}}j}} } \right)} }}} \\ {\frac{{{\mathbf{Z}}_{\text{NZJ}} {\mathbf{Z}}_{\text{DH}}^{\prime } }}{{\sqrt {\sum 2p_{{{\text{DH}}j}} \left( {1 - p_{{{\text{DH}}j}} } \right)} \sqrt {\sum 2p_{{{\text{NZJ}}j}} \left( {1 - p_{{{\text{NZJ}}j}} } \right)} }}} & {\frac{{{\mathbf{Z}}_{\text{NZJ}} {\mathbf{Z}}_{\text{NZJ}}^{\prime } }}{{\sum 2p_{{{\text{NZJ}}j}} \left( {1 - p_{{{\text{NZJ}}j}} } \right)}}} \\ \end{array} } \right] ,$$

where ${\mathbf{Z}}_{\text{DH}}$ and ${\mathbf{Z}}_{\text{NZJ}}$ are matrices containing centred genotype codes for all individuals from DH and NZJ populations, respectively, which were centred with breed-specific allele frequencies for all loci, *p*_DH*j*_ is the allele frequency for locus *j* in the DH population and *p*_NZJ*j*_ is the allele frequency for locus *j* in the NZJ population.

Two types of bivariate GREML models were implemented in the MTG2 [24] software using the calculated GRM and phenotypes.

#### MBSG model

This model is a bivariate, multi-breed, single GRM (MBSG) model. The bivariate model considers the phenotypes of DH and NZJ for the same trait as those from two different correlated traits and uses one GRM combining all markers. The model was as follows:

$$\left[ {\begin{array}{*{20}c} {{\mathbf{y}}_{\text{DH}} } \\ {{\mathbf{y}}_{\text{NZJ}} } \\ \end{array} } \right] = \left[ {\begin{array}{*{20}c} {1_{\text{DH}} } & 0 \\ 0 & {1_{\text{NZJ}} } \\ \end{array} } \right]\left[ {\begin{array}{*{20}c} {\mu_{\text{DH}} } \\ {\mu_{\text{NZJ}} } \\ \end{array} } \right] + \left[ {\begin{array}{*{20}c} {{\mathbf{W}}_{\text{DH}} } & 0 \\ 0 & {{\mathbf{W}}_{\text{NZJ}} } \\ \end{array} } \right]\left[ {\begin{array}{*{20}c} {{\mathbf{g}}_{\text{DH}} } \\ {{\mathbf{g}}_{\text{NZJ}} } \\ \end{array} } \right] + \left[ {\begin{array}{*{20}c} {{\mathbf{e}}_{\text{DH}} } \\ {{\mathbf{e}}_{\text{NZJ}} } \\ \end{array} } \right],$$

where ${\mathbf{y}}$ is a vector of phenotypes, *μ* is the trait mean, ${\mathbf{W}}$ is an incidence matrix linking observations in ${\mathbf{y}}$ to genetic effects in ${\mathbf{g}}$, and ${\mathbf{e}}$ is the residual. Genetic effects were assumed to be normally distributed as $$\left[ {\begin{array}{*{20}c} {{\mathbf{g}}_{\text{DH}} } \\ {{\mathbf{g}}_{\text{NZJ}} } \\ \end{array} } \right]\sim\,N\left( {0, {\mathbf{K}} \otimes {\mathbf{GRM}}} \right)$$ and $${\mathbf{K}} = \left[ {\begin{array}{*{20}c} {\sigma_{{g_{{\text{DH}}} }}^{2} } & {\sigma_{{g_{{\text{DH},\text{NZ}\text{J}}} }} } \\ {\sigma_{{g_{{\text{DH},\text{NZ}\text{J}}} }} } & {\sigma_{{g_{{\text{NZ}\text{J}}} }}^{2} } \\ \end{array} } \right]$$, where $\sigma_{{g_{{\text{DH}}} }}^{2}$ and $\sigma_{{g_{{\text{NZ}\text{J}}} }}^{2}$ are genetic variances in DH and NZJ populations, respectively, and $\sigma_{{g_{{\text{DH},\text{NZ}\text{J}}} }}$ is the genetic covariance between the breeds. The GRM fitted in this model were those computed based on SNPs in the 50k, TOP, COJO8, 50k + TOP and 50k + COJO8 sets for the empirical analysis. For the simulated scenarios, GRM based on 50k, TOP, TOP + RN, CAUSAL, NON CAUSAL and ALL sets were fitted.

#### MBMG model

This model is a bivariate, multi-breed, multi-GRM (MBMG) model and is an extension of MBSG, in which instead of a single multi-breed GRM, two separate multi-breed GRM (formed from two different marker sets) were fitted simultaneously. The model was as follows:

$$\begin{aligned} \left[ {\begin{array}{*{20}c} {{\mathbf{y}}_{\text{DH}} } \\ {{\mathbf{y}}_{\text{NZJ}} } \\ \end{array} } \right] & = \left[ {\begin{array}{*{20}c} 1 & 0 \\ 0 & 1 \\ \end{array} } \right]\left[ {\begin{array}{*{20}c} {\mu_{\text{DH}} } \\ {\mu_{\text{NZJ}} } \\ \end{array} } \right] + \left[ {\begin{array}{*{20}c} {{\mathbf{W}}_{{1{\text{DH}}}} } & 0 \\ 0 & {{\mathbf{W}}_{{1{\text{NZJ}}}} } \\ \end{array} } \right]\left[ {\begin{array}{*{20}c} {{\mathbf{g}}_{{1{\text{DH}}}} } \\ {{\mathbf{g}}_{{ 1 {\text{NZJ}}}} } \\ \end{array} } \right] \\ & \quad + \;\left[ {\begin{array}{*{20}c} {{\mathbf{W}}_{{2{\text{DH}}}} } & 0 \\ 0 & {{\mathbf{W}}_{{2{\text{NZJ}}}} } \\ \end{array} } \right]\left[ {\begin{array}{*{20}c} {{\mathbf{g}}_{{2{\text{DH}}}} } \\ {{\mathbf{g}}_{{2{\text{NZJ}}}} } \\ \end{array} } \right] + \left[ {\begin{array}{*{20}c} {{\mathbf{e}}_{\text{DH}} } \\ {{\mathbf{e}}_{\text{NZJ}} } \\ \end{array} } \right], \\ \end{aligned}$$

where subscripts 1 and 2 represents the first and second GRM fitted in the model, respectively, ${\mathbf{W}}_{1}$ and ${\mathbf{W}}_{2}$ are identical incidence matrices linking observations in ${\mathbf{y}}$ to the two genetic effects, ${\mathbf{g}}_{1}$ and ${\mathbf{g}}_{2}$. Here, genetic effects were also assumed to be normally distributed as:

$$\left[ {\begin{array}{*{20}c} {{\mathbf{g}}_{{1{\text{DH}}}} } \\ {{\mathbf{g}}_{{1{\text{NZJ}}}} } \\ \end{array} } \right]\sim\,N\left( {0,\varvec{ }{\mathbf{K}}_{1} \otimes {\mathbf{GRM}}1} \right),$$

$$\left[ {\begin{array}{*{20}c} {{\mathbf{g}}_{{2{\text{DH}}}} } \\ {{\mathbf{g}}_{{2{\text{NZJ}}}} } \\ \end{array} } \right]\sim\,N\left( {0,\varvec{ }{\mathbf{K}}_{2} \otimes {\mathbf{GRM}}2} \right),$$

with $${\mathbf{K}}_{1} = \left[ {\begin{array}{*{20}c} {\sigma_{{g_{{1\text{DH}}} }}^{2} } & {\sigma_{{g_{{1\text{DH},\text{NZ}\text{J}}} }} } \\ {\sigma_{{g_{{1\text{DH},\text{NZ}\text{J}}} }} } & {\sigma_{{g_{{1\text{NZ}\text{J}}} }}^{2} } \\ \end{array} } \right]$$ and $${\mathbf{K}}_{2} = \left[ {\begin{array}{*{20}c} {\sigma_{{g_{{2\text{DH}}} }}^{2} } & {\sigma_{{g_{{2\text{DH},\text{NZ}\text{J}}} }} } \\ {\sigma_{{g_{{2\text{DH},\text{NZ}\text{J}}} }} } & {\sigma_{{g_{{2\text{NZ}\text{J}}} }}^{2} } \\ \end{array} } \right]$$. For the empirical analysis, the sets of GRM fitted together in the MBMG model were 50k and TOP, 50k and COJO8. For the simulation analysis, the sets of GRM fitted together were TOP and 50k, TOP and NON CAUSAL, CAUSAL and NON CAUSAL.

For both the MBSG and MBMG models, residual terms were assumed to be normally distributed as ${\mathbf{e}}\sim\,N\left( {{\text{0}},{\mathbf{D}}\sigma_{\text{e}}^{2} } \right)$ in the empirical analysis, where *σ*_e_^2^ is the residual variance and ${\mathbf{D}}$ is a diagonal matrix that contains the inverse of dEDC, which were used as weights for the DRP. The dEDC in ${\mathbf{D}}$ were used to scale the residual of the animals in the model, such that animals with a high dEDC have effectively a small residual. For the analysis using simulated phenotypes, we assumed ${\mathbf{e}}\sim\,N\left( {{\text{0}},{\mathbf{I}}\sigma_{\text{e}}^{2} } \right)$ where ${\mathbf{I}}$ is an identity matrix.

For the multi-breed GP models described above, the reference population contained both DH and a subset (*n* = 476) of NZJ bulls. NZJ bulls were always used as validation individuals using fivefold cross-validation, where the NZJ bulls were randomly split into five sets of 119 individuals. The same validation sets were used for both the analyses using real and simulated data. The GEBV of bulls in a particular validation set were estimated based on the model obtained from the rest of the data, i.e. phenotypes of all DH bulls and the remaining 476 NZJ bulls. This was repeated five times (once for each validation set). In all models, accuracy of prediction was computed as the correlation between the GEBV of NZJ bulls in each validation set and their TBV, which in the analysis using real data were approximated by the DRP. The mean correlation across the five validation sets was used as the prediction accuracy.

To assess the benefit of combining both breeds in the reference population compared to only a single breed in the reference population, we implemented alternative models to MBSG and MBMG in which only DH (across-breed) or only NZJ (within-breed) bulls were included in the reference population. The single-breed equivalent to MBSG was:

$${\mathbf{y}} = {\bf 1}\upmu + {\mathbf{Wg}} + {\mathbf{e}},$$

where ${\mathbf{y}}$ is a vector of phenotypes for either DH (across-breed), in which case the model is referred to as across-breed, single-GRM (ABSG), or NZJ (within-breed), in which case the model is referred to as within-breed, single-GRM (WBSG). All other model parameters are as described under MBSG. The single-breed equivalent to MBMG was:

$${\mathbf{y}} = {\bf 1}\upmu + {\mathbf{W}}_{1} {\mathbf{g}}_{1} + {\mathbf{W}}_{2} {\mathbf{g}}_{2} + {\mathbf{e}},$$

where ${\mathbf{y}}$ is a vector of phenotypes for either DH (across-breed), in which case the model is referred to as across-breed, multiple-GRM (ABMG), or NZJ (within-breed), in which case the model is referred to as within-breed, multiple-GRM (WBMG). All other model parameters are as described under MBMG.

The models ABSG, ABMG, WBSG, and WBMG were fitted using both real and simulated phenotypes and NZJ bulls were the validation candidates. In the analyses of real data, the calculated accuracies of prediction, which were the correlations between DRP and GEBV, were scaled by the mean accuracy of the DRP. The dEDC for all NZJ were converted into reliabilities (*r*^2^) of DRP as $r^{2} = \frac{\text{dEDC}}{{{\text{dEDC}} + \frac{1}{{h^{2} }}}}$, where *h*^2^, in this case, is the heritability of stature in NZJ. The mean *r*^2^ for the validation candidates (NZJ) was equal to 0.76.

## Results

### Empirical phenotypes

#### Estimated genetic parameters

In the analysis using DRP for stature, we estimated *h*_DRP_^2^ for both DH and NZJ using the MBSG and MBMG models, which in this case reflect the proportion of the explained variance of DRP. These estimates are in Table 1. The estimated *h*_DRP_^2^ for DH and NZJ were similar between the bivariate multi-breed models and their equivalent univariate models (results not shown). Thus, estimates of *h*_DRP_^2^ obtained with the MBSG model were similar to those obtained with the ABSG model for DH, and WBSG for NZJ. Likewise, estimates of *h*_DRP_^2^ obtained with the MBMG model were similar to those obtained with ABMG for DH and WBSG for NZJ. In general, estimated *h*_DRP_^2^ were lower for NZJ than DH, which reflected the smaller number of NZJ individuals in the reference population and their lower mean dEDC (17). The mean of dEDC for the DH bulls was equal to 52.

**Table 1:** Estimated heritability (*h*_DRP_^2^) obtained by fitting different genomic relationship matrices (GRM) formed from different sets of markers and the corresponding genetic correlation (*r*_g_) between Dutch Holstein (DH) and New Zealand Jersey (NZJ) breeds

GRM fitted	*h*_DRP_^2^ DH	*h*_DRP_^2^ NZJ	Estimated *r*_g_ (DH vs. NZJ)
MBSG			
50k	0.97 (0.00)	0.71 (0.02)	0.22 (0.17)
TOP	0.26 (0.01)	0.27 (0.01)	0.41 (0.15)
COJO8	0.75 (0.01)	0.38 (0.02)	0.44 (0.11)
50k + TOP	0.97 (0.00)	0.71 (0.02)	0.25 (0.17)
50k + COJO8	0.97 (0.00)	0.71 (0.03)	0.29 (0.17)
MBMG			
TOP and 50k	0.25 (0.01) and 0.71 (0.01)	0.11 (0.01) and 0.61 (0.02)	0.64 (0.17) and 0.16 (0.19)
COJO8 and 50k	0.26 (0.01) and 0.70 (0.01)	0.17 (0.02) and 0.57 (0.02)	0.88 (0.14) and 0.21 (0.19)

Standard error of estimates are given between parentheses. In the multi-breed, single-GRM model (MBSG), a single multi-breed GRM was fitted in a prediction model, while in the multi-breed, multiple-GRM model (MBMG), two separate multi-breed GRM, formed from different SNP sets, were fitted simultaneously in a prediction model

When the GRM based on the TOP 133 SNPs was fitted in MBSG, estimated *h*_DRP_^2^ were low, i.e. 0.26 and 0.27 for DH and NZJ, respectively. However, when using the GRM based on the 357 COJO8 SNPs, estimated *h*_DRP_^2^ were significantly higher, i.e. 0.75 and 0.38 for DH and NZJ, respectively. Estimated *h*_DRP_^2^ were highest with the 50k GRM, i.e. 0.97 and 0.71 for DH and NZJ, respectively. *h*_DRP_^2^ did not increase further by adding either the TOP or COJO8 SNPs to the 50k set. Total *h*_DRP_^2^ estimated when fitting two separate GRM simultaneously in the MBMG model did not differ significantly from those obtained by fitting only the 50k GRM. For example, for DH, the TOP and COJO8 SNPs fitted as the first GRM resulted in estimated *h*_DRP_^2^ of 0.25 and 0.26, respectively, while the 50k SNPs fitted as the second GRM resulted in an *h*_DRP_^2^ of about 0.70.

Estimated *r*_g_ for stature between the DH and NZJ breeds, which can be interpreted as the correlation between the breeding values of individuals from the two breeds, are also in Table 1. With the MBSG model, in which a single GRM was fitted in a bivariate model, *r*_g_ were higher when only the pre-selected SNPs (TOP or COJO8) were fitted in the model compared to when the 50k or 50k plus pre-selected SNPs were fitted. However, given the large standard errors of the *r*_g_, the differences in estimated *r*_g_ between the different GRM were not significant. With the MBMG model, estimated *r*_g_ resulting from the pre-selected SNP sets were significantly higher than those obtained with MBSG. For the TOP SNPs, *r*_g_ increased from 0.41 when fitted alone in MBSG to 0.64 when fitted together with the 50k SNPs in MBMG. For the COJO8 SNPs, *r*_g_ increased from 0.44 when fitted alone in the model, to 0.88 when fitted together with the 50k SNPs in MBMG. While the estimated *r*_g_ due to pre-selected SNPs increased when fitted simultaneously with the 50k SNPs in MBMG, the estimated *r*_g_ obtained for the 50k set did not change significantly, but tended to decrease compared to that obtained when the 50k SNPs were fitted alone in MBSG. In general, estimated *r*_g_ between breeds were much higher for the pre-selected SNPs than for the 50k or 50k plus pre-selected SNPs.

### Accuracy of genomic breeding values

**Fig. 1:**
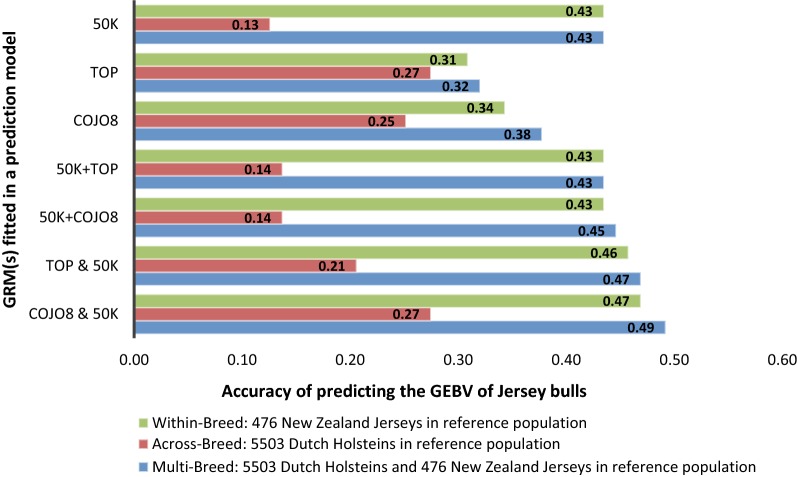
Accuracy of predicting genomic breeding values (GEBV) for stature of New Zealand Jersey (NZJ) bulls from a reference population consisting of only NZJ or only Dutch Holstein (DH) or a combination of NZJ and DH bulls

The accuracy of GEBV for the NZJ bulls using single or multi-breed reference populations is shown in Fig. 1. When using a single GRM and a NZJ reference population (WBSG model), accuracies of prediction were lower using the pre-selected marker sets (0.31 for the TOP and 0.34 for the COJO8 sets) than using the unselected 50k SNPs (0.43). Accuracy did not change by adding either the TOP or COJO8 SNPs to the 50k set. When using two separate GRM (WBMG model), accuracies increased slightly compared to a single GRM, i.e. 0.46 when fitting the TOP and 50k SNPs in separate GRM, and 0.47 when fitting the COJO8 and 50k SNPs in separate GRM.

When using a single GRM and a DH reference population (ABSG model), the highest prediction accuracies were obtained using the pre-selected marker sets, i.e. 0.27 when the TOP SNPs were fitted and 0.25 when the COJO8 SNPs were fitted. With the unselected marker sets (50k, 50k + TOP, 50k + COJO8), accuracies were low (~ 0.14). When using two separate GRM (ABMG model), accuracies were lower when fitting TOP and 50k SNPs in separate GRM (0.21) compared to the model with only the TOP SNPs fitted (0.27). Fitting COJO8 and 50k SNPs in separate GRM improved prediction accuracy slightly (0.27) compared to fitting only the COJO8 SNPs (0.25).

When using a single GRM and combined reference population of DH and NZJ bulls (MBSG model), prediction accuracies did not differ much from those obtained by using only NZJ in the reference population for the 50k, 50k + TOP and 50k + COJO8 sets. For the scenarios with pre-selected markers, accuracy of prediction increased compared to when only the Jersey bulls were included in the reference population. For the TOP and COJO8 sets, prediction accuracies increased from 0.31 to 0.32 and from 0.34 to 0.38, respectively, with a multi-breed reference population (MBSG) compared to only NZJ bulls (WBSG). When using two separate GRM in the MBMG model, prediction accuracies were always higher compared to the model with only one GRM or to the model with only NZJ in the reference population. For example, TOP and 50k GRM fitted in the MBMG model resulted in a prediction accuracy of 0.47, and COJO8 and 50k GRM fitted in the MBMG model resulted in an accuracy of 0.49.

### Simulated phenotypes

#### Estimated genetic parameters

In the simulation study, a quantitative trait with an *h*^2^ of 0.8 was simulated in the DH and NZJ populations. The estimates of *h*^2^ are in Table 2.

**Table 2:** Estimated heritability (*h*^2^) in Dutch Holstein (DH) and New Zealand Jersey (NZJ), and the estimated genetic correlation between breeds (*r*_g_) estimated using the different genomic relationship matrices (GRM) and simulated phenotypes (100 replicates)

GRM fitted	*h*^2^ DH	*h*^2^ NZJ	*r*_g_
*Simulated r*_*g*_ *=* *1*			
MBSG (1 GRM fitted in a bivariate model)			
ALL	0.80 (0.01)	0.76 (0.06)	0.98 (0.19)
50k	0.79 (0.01)	0.76 (0.07)	0.67 (0.18)
TOP	0.44 (0.03)	0.38 (0.06)	0.85 (0.05)
TOP + RN	0.48 (0.03)	0.44 (0.05)	0.77 (0.07)
CAUSAL	0.80 (0.02)	0.76 (0.02)	1.00 (0.00)
NON CAUSAL	0.77 (0.01)	0.71 (0.20)	0.20 (0.20)
MBMG (2 separate GRM fitted simultaneously in a bivariate model)			
TOP and 50k	0.37 (0.04) and 0.41 (0.04)	0.29 (0.05) and 0.48 (0.07)	1.01 (0.03) and 0.98 (0.21)
TOP and NON CAUSAL	0.37 (0.04) and 0.41 (0.03)	0.31 (0.05) and 0.47 (0.07)	1.02 (0.03) and 0.19 (0.22)
CAUSAL and NON CAUSAL	0.80 (0.02) and 0.00 (0.00)	0.76 (0.04) and 0.00 (0.02)	1.01 (0.01) and NA
*Simulated r*_*g*_ *=* *0.5*			
MBSG (1 GRM fitted in a bivariate model)			
ALL	0.80 (0.01)	0.80 (0.06)	0.50 (0.18)
50k	0.79 (0.01)	0.79 (0.06)	0.34 (0.18)
TOP	0.44 (0.03)	0.39 (0.05)	0.43 (0.12)
TOP + RN	0.48 (0.03)	0.44 (0.05)	0.41 (0.11)
CAUSAL	0.80 (0.01)	0.80 (0.02)	0.51 (0.05)
NON CAUSAL	0.78 (0.01)	0.79 (0.06)	0.11 (0.18)
MBMG (2 separate GRM fitted simultaneously in a bivariate model)			
TOP and 50k	0.37 (0.03) and 0.43 (0.04)	0.31 (0.05) and 0.49 (0.07)	0.52 (0.11) and 0.53 (0.21)
TOP and NON CAUSAL	0.38 (0.03) and 0.41 (0.03)	0.31 (0.05) and 0.48 (0.07)	0.52 (0.12) and 0.12 (0.21)
CAUSAL and NON CAUSAL	0.80 (0.01) and 0.00 (0.00)	0.80 (0.02) and 0.00 (0.02)	0.51 (0.05) and NA
*Simulated r*_*g*_ *=* *0.25*			
MBSG (1 GRM fitted in a bivariate model)			
ALL	0.80 (0.01)	0.80 (0.06)	0.27 (0.19)
50k	0.79 (0.01)	0.79 (0.06)	0.18 (0.18)
TOP	0.44 (0.03)	0.39 (0.05)	0.22 (0.13)
TOP + RN	0.48 (0.03)	0.44 (0.05)	0.22 (0.12)
CAUSAL	0.80 (0.01)	0.80 (0.02)	0.26 (0.06)
NON CAUSAL	0.78 (0.01)	0.76 (0.06)	0.06 (0.18)
MBMG (2 separate GRM fitted simultaneously in a bivariate model)			
TOP and 50k	0.37 (0.03) and 0.43 (0.04)	0.31 (0.05) and 0.49 (0.07)	0.27 (0.14) and 0.29 (0.21)
TOP and NON CAUSAL	0.38 (0.03) and 0.41 (0.03)	0.31 (0.05) and 0.48 (0.07)	0.27 (0.13) and 0.08 (0.21)
CAUSAL and NON CAUSAL	0.80 (0.01) and 0.00 (0.00)	0.80 (0.02) and 0.00 (0.03)	0.26 (0.06) and NA

Simulated heritability for the trait was 0.8 and simulated *r*_g_ between breed were 1, 0.5 and 0.25

In general, estimated *h*^2^ were lower in NZJ than in DH, as was also observed for a real phenotype. With a simulated *r*_g_ of 1 for the trait in DH and NZJ, the unselected marker sets (50k, NON CAUSAL, ALL) captured almost the entire *h*^2^ in DH, ranging from 0.77 to 0.8, while these values were slightly lower in NZJ ranging from 0.71 to 0.76. The TOP SNPs resulted in an estimated *h*^2^ of 0.44 in DH and 0.38 in NZJ. Adding 150 randomly chosen non-causal SNPs, representing random noise, to the TOP (TOP + RN) set resulted in increased *h*^2^, i.e. 0.48 in DH and 0.44 in NZJ. When the TOP SNPs were fitted together with either the 50k or NON CAUSAL sets in MBMG, estimated *h*^2^ using the TOP SNPs decreased to 0.37 in DH and ~ 0.30 in NZJ. The CAUSAL SNPs resulted in an estimated *h*^2^ of 0.8 in DH, both when fitted alone or together with NON CAUSAL SNPs. The NON CAUSAL SNPs resulted in an *h*^2^ of 0.76, when fitted with the CAUSAL SNPs. In general, there was no significant difference in the percentage of *h*^2^ captured between the scenarios with a simulated *r*_g_ of 1 and those with a simulated *r*_g_ of 0.5 or 0.25 (Table 2).

Estimated *r*_g_ using simulated phenotypes with a simulated *r*_g_ of 1 are also in Table 2. The estimated *r*_g_ obtained by fitting the CAUSAL GRM in MBSG was equal to 1. When all the SNPs (CAUSAL + NON CAUSAL) were fitted in MBSG, the estimated *r*_g_ was equal to 0.98 but decreased to 0.67 when only the 50k SNPs were fitted. When the TOP SNPs were fitted, the estimated *r*_g_ was equal to 0.85 and decreased to 0.77 when some 150 random noise SNPs were added to the TOP SNPs (TOP + RN). In general, estimates of *r*_g_ were more precise when the causal markers were isolated from the non-causal markers in a separate GRM (MBMG model), but also when all the causal markers were present in the GRM. In general, estimated *r*_g_ were proportional to the simulated *r*_g_ (Table 2).

### Accuracy of genomic breeding values

#### Simulated genetic correlation of 1 between breeds

**Fig. 2:**
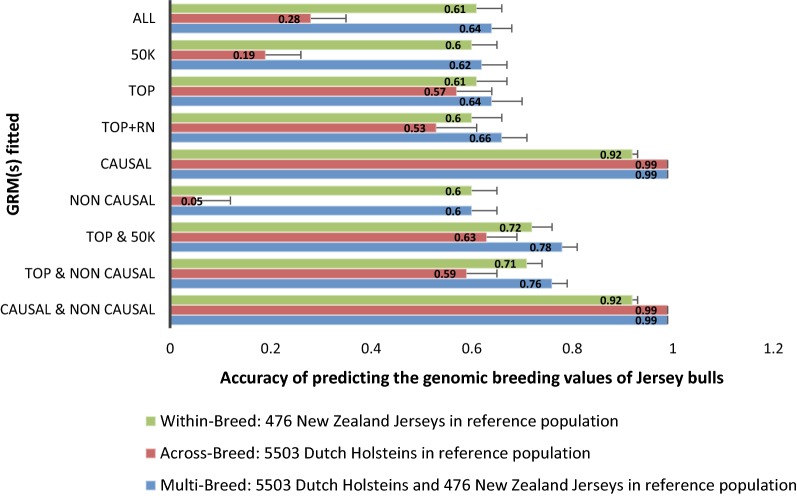
Accuracy with standard errors of predicting the genomic breeding values (GEBV) of Jersey bulls from a reference population made of only Jersey or only Holstein or a combination of Holstein and Jersey bulls. Simulated genetic correlation between the breeds was 1

In the WBSG model, the unselected marker sets (50k, NON CAUSAL, and ALL) resulted in accuracies of ~ 0.60 (Fig. 2). The TOP and TOP + RN sets also gave accuracies of ~ 0.60, although they consisted only of a fraction of the SNPs in the unselected marker sets. Except when fitting the CAUSAL SNPs in within-breed, single-GRM model, accuracies were significantly higher when two separate GRM were fitted simultaneously rather than those in which only a single GRM was fitted.

In the ABSG model, in which a DH reference population was used to predict NZJ GEBV, accuracies were much lower than those obtained in the within-breed single-GRM model for the unselected marker sets. However, accuracies were only slightly lower in ABSG for the TOP and TOP + RN SNP sets than those obtained in the within-breed single-GRM model. The only scenarios in which across-breed prediction outperformed within-breed prediction were the scenarios in which the CAUSAL SNPs were fitted in ABSG and the CAUSAL and NON CAUSAL SNPs were fitted simultaneously in ABMG, which reflects the larger reference population of DH than of NZJ and an *r*_g_ of 1. In general, across-breed prediction accuracies were by far higher when the pre-selected marker sets were used for prediction compared to when the unselected marker sets (50k, NON CAUSAL, and ALL) were used. Except when fitting the CAUSAL SNPs in ABSG, across-breed prediction accuracies were significantly higher with the ABMG model than with the ABSG model.

Combining DH and NZJ bulls in the reference population to obtain NZJ GEBV (multi-breed prediction) resulted in slightly better prediction accuracies than when only NZJ were used in the reference population. As also observed with empirical phenotypes, the highest accuracies were obtained in MBMG, except in the unrealistic case that all causal SNPs could be identified with 100% accuracy and were all fitted in the MBSG model.

### Simulated genetic correlation of 0.5 and 0.25 between breeds

**Fig. 3:**
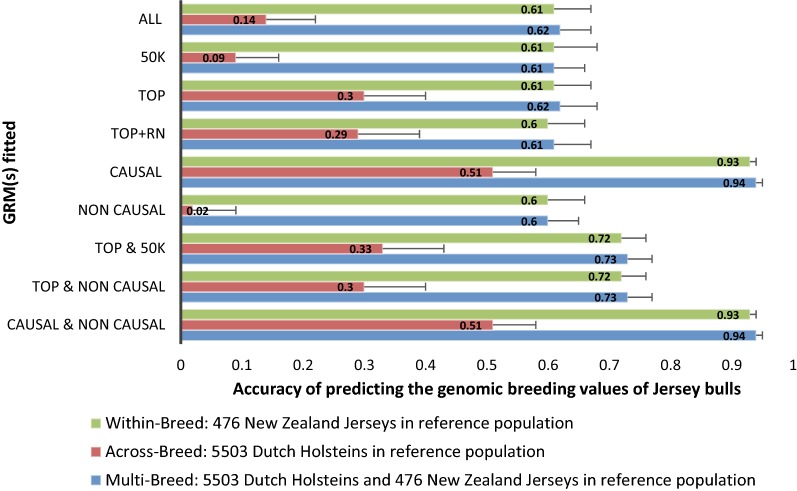
Accuracy with standard error of predicting the genomic breeding values (GEBV) of Jersey bulls from a reference population made of only Jersey or only Holstein or a combination of Holstein and Jersey bulls. Simulated genetic correlation between breed was 0.5

**Fig. 4:**
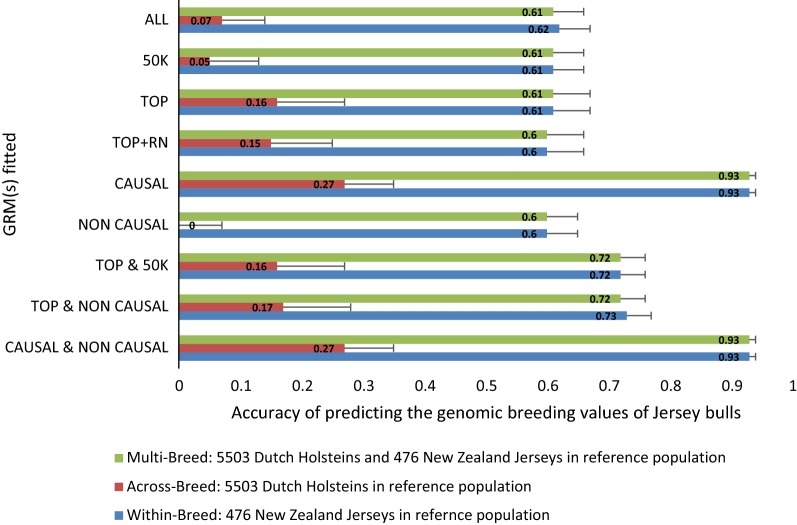
Accuracy with standard error of predicting the genomic breeding values (GEBV) of Jersey bulls from a reference population made of only Jersey or only Holstein or a combination of Holstein and Jersey bulls. Simulated genetic correlation between breed was 0.25

Figures 3 and 4 show the prediction accuracies of the simulation with a *r*_g_ of 0.5 and 0.25 respectively, between DH and NZJ. In general, prediction accuracies of GEBV for the NZJ bulls were lower with a simulated *r*_g_ between DH and NZJ of 0.5 and 0.25 than with an *r*_g_ of 1, especially when only DH were included in the reference population. In almost all cases, accuracies were higher in scenarios in which two separate GRM were fitted simultaneously than those in which only a single GRM was fitted. One exception was when the CAUSAL GRM was fitted alone. In across-breed prediction, when only the DH reference population was used to obtain GEBV for NZJ, the accuracy of prediction was about 50% lower when the simulated *r*_g_ was 0.5 instead of 1 and 75% lower when the simulated *r*_g_ was 0.25 instead of 1. With a simulated *r*_g_ of 0.5 and 0.25, combining DH and NZJ in the reference population did not improve prediction accuracy compared to a reference population including only NZJ individuals, although the combined reference population was more than 10 times larger than the NZJ reference population.

## Discussion

The objective of this study was to test the performance of a multi-breed, multi-GRM (MBMG) model in terms of estimated genetic parameters and accuracy of predicting GEBV of individuals from a numerically small population. The results of our analyses using both real and simulated phenotypes demonstrated the superiority of the MBMG model in terms of prediction accuracy in comparison with a single GRM fitted in within-, across-, and multi-breed prediction models. The MBMG model also outperformed a within-(WBMG) and across-breed (ABMG) GP model with two separate GRM fitted simultaneously. Three main distinguishing properties of MBMG are: (1) prioritisation of pre-selected functional markers over other types of markers, (2) use of the unselected markers to account for RGV_m in such a way that they do not dilute the effect of the pre-selected functional markers, and (3) appropriate weighting of information provided by each breed in the reference population by their *r*_g_ with the validation breed.

Prioritisation of known QTL or pre-selected functional markers over other types of markers for the estimation of breeding values not an entirely new concept, and was already the main idea behind marker-assisted selection (MAS) [25, 26]. With MAS, pre-selected significant markers from a genome-wide association study (GWAS) are used to predict breeding values for a certain trait. One major pitfall of MAS for complex traits was that the pre-selected significant markers accounted for only a small fraction of the total genetic variance for the traits of interest [25]. As a result, there was little to no gain in using (only) MAS over phenotypic selection [26], and therefore most applications have focussed on GP using many markers across the genome [27]. Even in this genomic selection era, a number of studies have advocated the prioritization of causal regions in GP models, especially as a result of the increasing availability and interest in the use of whole-genome sequence (WGS) data in breeding [28, 29]. For example, Brøndum et al. [28] showed that when significant SNPs from a GWAS using WGS data are added to a low-density SNP panel (50k), and used for GP, accuracy of within-breed prediction can be increased. Also, van den Berg et al. [29] showed that adding SNPs that are near QTL peaks in a GWAS to a 50k panel improved the accuracy of within- and multi-breed GP as compared to the use of a 50k panel only. In the absence of additional markers, using only pre-selected markers has been shown to result in little or no advantage for within-breed GP [30], which was confirmed in this study. In general, when we fitted only the pre-selected (TOP and COJO8) SNPs in a GP model and using real phenotypes, accuracies of within-breed GP were lower compared to when we fitted the unselected SNP sets (50k, 50k + TOP, 50k + COJO8) (Fig. 1). One reason could be that, for within-breed GP in which the training set is the same breed as the validation set, the unselected markers can still explain part of the genetic variance due to LD between markers and QTL that are not yet identified (Table 1). Since long-range LD is present between markers and QTL within breed, it is less important to identify the true causal markers for within-breed GP.

Across breeds, the LD phase between markers and QTL is not necessarily consistent [7, 31] Consequently, using non-causal markers that do not tag the QTL through LD, may result in poor across-breed GP accuracies, as was observed in this study for the unselected SNP sets and by others [3–5, 32]. This is mainly because the estimated effect of a non-causal marker is proportional to the extent of its LD with a QTL [33]. Thus, if LD phase between the non-causal marker and QTL is different between breeds, then the non-causal marker would have different estimated effects in different breeds, which is not accounted for in the prediction model. For across-breed GP, it is important to identify the QTL or markers close to QTL and use them for prediction [31]. Our results from the analyses of real and simulated phenotypes showed that when markers are pre-selected based on their causal effects and used for across-breed GP, the accuracy of prediction is significantly higher than when all markers, including non-causal or unselected markers are used. The obtained accuracies of across-breed GP agree with those of Saatchi and Garrick [32] who found that across-breed genomic prediction resulted in accuracies close to 0, except for some traits due to the segregation of common large-effect QTL with conserved linkage phase among the different breeds.

Pre-selection of markers based on their potential causal effect is not trivial [34]. This is especially the case when pre-selection of potential causal markers is carried out for traits with a complex genetic architecture within a single population using GWAS. In those situations, the power of GWAS methods to precisely identify the causative mutations for the traits of interest is hindered by the high relatedness between individuals, strong and long range LD between QTL and many non-causal markers within the genome, many QTL with very small effect sizes, low allele frequency of the QTL, and small sample size [35]. In the current study, we benefitted from the results of a large international meta-GWAS [19], in which some of these limiting factors, such as small sample size, use of a single population, confounding effect of LD and family relationships were overcome by the use of multiple populations consisting of eight cattle breeds form different countries. Nevertheless, it is not guaranteed that all pre-selected SNPs are truly causal, but the ones that are not false positives are at least in high LD with and located close to the true causal loci. The large power in the meta-GWAS to identify the potential causal markers that we used in our study can probably explain the improved prediction accuracies in the across- and multi-breed GP scenarios using pre-selected markers.

Another possible pitfall of pre-selection of markers based on the results of GWAS is the likely event of false positive findings. To investigate the effect of including random noise on prediction accuracy, our simulation study included a scenario (TOP + RN) in which 150 false positive SNPs were added to 133 causal SNPs. For across-, and multi-breed GP, our results showed very little to no effect of adding the random noise (53% of the SNPs) to the causal SNPs on prediction accuracy. When the non-causal markers outweigh very much the number of true causal markers in a set of markers, as was the case in the 50k scenario, accuracy of prediction becomes considerably lower than when using only the true causal markers. Also Wang et al. [36] showed that reducing the number of non-causal markers from 12,000 to 1000 that were added to a set of 10 true causal markers resulted in higher prediction accuracy. In practise, a much lower proportion of false positive markers can be assumed, e.g. a 5% false discovery rate is commonly used. Our results showed that it is unlikely that pre-selected false positive markers (less than 53% of all pre-selected markers) will have a significant negative effect on prediction accuracy.

In practice, pre-selected markers from a GWAS using whole-genome sequence data may not be found on the traditional 50k SNP chip. In order to use the pre-selected SNPs in routine genomic evaluations with the MBMG model, the pre-selected SNPs may have to be imputed in the populations of interest. However, some pre-selected SNPs, especially those with a low MAF, could be imputed with relatively low accuracy, which could, in turn, negatively impact the performance of the MBMG model. Still, the impact will most likely be small, given that, in general, SNPs with a low MAF explain only a small proportion of the genetic variance. One way that the imputation error of markers could be accounted for in GP is to use dosage scores rather than genotype calls, to calculate the GRM. A more practical solution for the breeding industry is to add known QTL or pre-selected SNPs to custom chips, as this will result in more accurate genotypes.

The MBMG model uses the unselected markers to explain RGV_m that is not explained by the pre-selected markers. Thus, instead of fitting only the pre-selected markers, we fitted both pre-selected and unselected markers in two separate GRM, by using the multi-breed reference population. In this way, the model benefits from improved tagging of QTL by the pre-selected markers across breeds and, at the same time, benefits from additional genetic variance explained by the unselected markers within each breed. If all the QTL that underlie a trait are identified with 100% accuracy, there would be no need to include other markers in the prediction model, since the QTL could explain 100% of the genetic variance (the CAUSAL scenarios in our simulations). However, in practice it is unlikely to identify all the QTL for complex traits. For example, the top SNPs in the 163 QTL identified in the large meta-GWAS for stature explained only 13.8% of phenotypic variance for the trait [19]. This shows the importance of including multiple GRM in the MBMG model to maximise the proportion of total genetic variance explained within breed.

Appropriate weighting of the information provided by each breed in the reference population by their *r*_g_ with the validation breed is an important feature of the MBMG model. Multi-breed GP can be implemented by simply pooling the information of different breeds in a univariate GP model, effectively assuming a *r*_g_ of 1 between the breeds in the reference population and between the reference and validation breeds. Assuming a *r*_g_ of 1 between breeds, especially between distantly related breeds such as Holstein and Jersey, can result in little to no advantage or even poorer prediction accuracies, as compared to a within-breed prediction model [37]. The MBMG model is a multi-trait model in which the phenotypes from different breeds are treated as those from different but correlated traits [37–39]. The expectation is that multi-trait modelling only uses information from another breed that will improve accuracy or, in the worst case, will not affect the accuracy, but it will not decrease the accuracy either [39].

In the traditional multi-trait models used for multi-breed GP (MBSG), an implicit assumption that is made is that *r*_g_ is the same across the entire genome. Due to differences in genetic background and breeding practices between breeds, traits might have evolved differently [40]. In such cases, it is expected that marker effects, including the effect of causal markers, have different covariance structures in the different breeds. In MBMG, we assumed that the *r*_g_ between breeds at the causal markers can be different from the *r*_g_ between breeds at the unselected markers. We believe that the assumption of different *r*_g_ for different regions in the genome is one of the factors that make MBMG superior to MBSG in both our empirical and simulation analyses (Figs. 1, 2, 3).

In addition to accounting for the RGV_m, fitting a second GRM made from the unselected markers in MBMG allowed for a more precise estimation of the *r*_g_ between breeds at the pre-selected markers compared to fitting only the pre-selected markers in MBSG (Tables 1, 2). For example, in the scenario with a simulated *r*_g_ of 1 between DH and NZJ, the estimated *r*_g_ at the TOP SNPs was 0.88 when only the TOP SNPs were fitted in the model (MBSG). However, by fitting the 50k GRM as a separate component in MBMG, the estimated *r*_g_ at the TOP SNPs was exactly 1, as simulated. The difference in the estimate of *r*_g_ at the pre-selected markers between MBSG and MBMG can probably be explained as follows: when the pre-selected markers are fitted alone in MBSG, they also explain RGV_m due to long-range LD within the breeds. However, when the pre-selected markers are fitted together with other markers in separate GRM (MBMG), the pre-selected markers account for the genetic variance that is mainly due to the pre-selected markers, and the RGV_m is explained by other markers in the second GRM. The covariance between breeds (the numerator) at the pre-selected markers remains similar between both models (MBSG and MBMG), since genetic covariance depends only on the conserved LD phase between breeds, which does not change between models (results not shown). Thus, the inflated genetic variance within breeds (the denominators) explained by the pre-selected markers in MBSG results in a lower estimated *r*_g_ at the pre-selected markers using MBSG than using MBMG. However, even in MBSG, we observed higher estimates of *r*_g_ at the pre-selected markers than at the unselected markers. This is because the precision of estimating *r*_g_ between breeds using markers depends on how accurately the marker-based relationships describes the relationships at the causal markers [18]. Hence, the estimated *r*_g_ is more precise for causal markers than for non-causal markers.

The MBMG model follows a similar principle since it can be implemented in Bayesian models, for example, in the BayesRC method [41] or the so-called multitask Bayesian learning model [42, 43]. Similar to MBMG, BayesRC incorporates prior biological knowledge in the prediction model by assigning different markers into different classes, for instance, a pre-selected marker class and unselected marker class, or even more classes. One of the differences between MBMG and BayesRC is that in MBMG, we assumed a priori that the effects of all markers in a certain class follow a normal distribution whereas in BayesRC, for each class of markers, there is a mixture of four normal distributions, which allows for pre-selected markers to have a large effect, an average effect, a small effect, or no effect (false positive). In addition to allowing for different effect sizes of markers and QTL as in BayesRC, the multitask Bayesian variable selection model for multi-breed GP allows for markers associated with a breed specific QTL to have a large effect in one breed and a small effect in another breed. Calus et al. [43] showed that, compared to a multi-trait GREML model (MBSG) that assumes a single *r*_g_ across the genome, the multitask Bayesian variable selection model had similar accuracies of prediction for traits with a relatively high estimated *r*_g_ between Holstein and Jersey, but outperformed the MBSG model for traits with a very low estimated *r*_g_ between Holstein and Jersey. Our expectation is that Bayesian models will be more suited than MBMG for prediction of traits that are influenced by a few QTL with large effects and many additional smaller QTL, such as milk fat percentage with the *DGAT1* QTL. Otherwise, when traits are influenced by many QTL with small effects, we expect the MBMG and the Bayesian models to perform similarly. This is mainly because, in such case, the assumption of the GREML model that the effect sizes of QTL or markers linked to QTL follow a normal distribution holds.

## Conclusions

We presented a multi-breed multi-GRM model, MBMG, for genomic selection in numerically small breeds. The key features of MBMG are: (1) appropriate use of information on known QTL or pre-selected markers; (2) explanation of RGV_m by the unselected markers; and (3) use of the genetic correlation between reference and validation breeds to weigh the contribution of each breed in the reference population to the accuracy of prediction. Our proposed MBMG model can be applied to any case in which some form of prior information on markers exist, e.g. genome annotation information, experimental evidence, etc. By using this model, we are able to better combine information from numerically small and large breeds to improve prediction accuracy for the numerically small breed using both pre-selected and unselected markers. The MBMG model, together with the availability of whole-genome sequence data and accurate marker pre-selection strategies, can result in more accurate genomic prediction in numerically small breeds and thus improve their rate of genetic gain.
